# Genome-wide analysis of the R2R3-MYB family reveals potential regulators of lignin and tricin metabolism in the model grass *Setaria viridis*

**DOI:** 10.1007/s00438-026-02355-w

**Published:** 2026-02-07

**Authors:** Giovanni Victorio Cerruti, Lucas Xavier da Cunha, Alice Cristine Cursino, Marcella Siqueira Simões, Igor Cesarino

**Affiliations:** 1 Departamento de Botânica, Instituto de Biociências, Universidade de São Paulo, Rua do Matão, 277, São Paulo, 05508-090 Brazil; 2 Synthetic and Systems Biology Center, InovaUSP, Avenida Professor Lucio Martins Rodrigues, 370, São Paulo, 05508-020 Brazil

**Keywords:** Gene expression, Grasses, Lignin, R2R3-MYBs, Tricin, *Setaria viridis*

## Abstract

R2R3-MYBs constitute one of the largest families of plant transcription factors and several members are involved in the regulation of distinct branches of the phenylpropanoid pathway, acting as activators or repressors of the biosynthesis of a plethora of specialized metabolites. Although R2R3-MYBs have been extensively characterized as regulators of lignin deposition in distinct cellular contexts and species, still little is known about their roles in regulating specific aspects of grass lignification. Here, we report on the genome-wide characterization of the R2R3-MYB family in the model grass *Setaria viridis* and identification of members potentially involved in the regulation of lignin/tricin metabolism. A total of 132 genes encoding R2R3-MYBs were found in *S. viridis*, which clustered in 43 well-supported subgroups. Comprehensive in silico expression, co-expression, and RT-qPCR analyses allowed the identification of 4 candidate *SvMYB*s that showed (i) similar expression profiles to that observed for lignin biosynthetic genes in a set of different organs/conditions of *S. viridis*; (ii) similar expression patterns to that of lignin biosynthetic genes along the *S. viridis* elongating internode; (iii) co-expression with several phenylpropanoid- and lignin-related genes in public transcriptomic databases; (iv) high expression levels in the top of the *S. viridis* elongating internode, a tissue undergoing active lignification. Three of these SvMYBs activated the promoters of lignin and tricin biosynthetic genes in transactivation assays using tobacco protoplasts. Altogether, our results suggest that these three transcription factors control grass-specific aspects of lignin deposition and further studies might confirm their ability to control lignin deposition and tricin metabolism in *S. viridis*.

**Supplementary Information:**

The online version contains supplementary material available at 10.1007/s00438-026-02355-w.

## Introduction

R2R3-MYBs constitute one of the largest families of plant transcription factors and regulate diverse aspects of plant growth and development, as well as responses to biotic and abiotic stresses (Dubos et al. 2010; Pratyusha and Sarada 2022). Several R2R3-MYBs are involved in the regulation of distinct aspects of the phenylpropanoid metabolism, acting as both activators or repressors of the biosynthesis of specialized metabolites including flavonoids, phenolic acids, and stilbenes, as well as of the deposition of lignin during the formation of SCWs and apoplastic barriers such as Casparian strips (Behr et al. 2019; Figueiredo et al. 2020; Liu et al. 2015; Ma and Constabel 2019). For instance, a set of 13 MYBs form a multi-leveled feed-forward loop regulatory network that controls the balance between lignification and suberization in Arabidopsis roots (Xu et al. 2022). In cucumber, CsMYB36 is the orthologue of Arabidopsis MYB36, which is a master regulator of Casparian strip formation in roots (Kamiya et al. 2015), and regulates lignin deposition during the formation of the “neck strip” in glandular trichomes (Hao et al. 2024). Structurally, R2R3-MYB proteins have two adjacent N-terminal DNA-binding domain repeats (the R2R3 MYB domain) and a variable C-terminal transcriptional regulation domain, which can have either an activation or a repression activity (Dubos et al. 2010; Liu et al. 2015). Pioneer genome-wide analyses in Arabidopsis have classified these transcription factors into 23 subgroups based on the conserved amino acid sequence motifs found in their most C-terminal MYB domain, although for many members no motif was found to group them (Dubos et al. 2010; Stracke et al. 2001). Subsequent work has shown that most of these subgroups were conserved in other plants, including both eudicots and monocots, but also new subgroups absent in Arabidopsis were found in some plant species/groups (Li et al. 2016; Shelton et al. 2012; Soler et al. 2015; Yang et al. 2021a, b). Given that MYBs from the same subgroup were frequently shown to have similar biological functions (Agarwal et al. 2016; Hao et al. 2024; Shen et al. 2012; Zhong et al. 2015), functional inference based on orthology/homology relationships is a key strategy to select candidate R2R3-MYBs with unknown function for further characterization.

Grasses are one of the most economically important groups of plants, constituting a prominent source of food, feed, and biomass for the production of bioproducts in biorefineries (Burton and Fincher 2014; Cesarino et al. 2016). Grasses differ considerably from eudicots in terms of vascular patterning and SCW/lignin structure and composition, suggesting grass-specific molecular mechanisms underlying that are not found in eudicots and whose knowledge cannot be extrapolated from data obtained with eudicot species. For instance, the flavone tricin is a noncanonical lignin monomer found in grasses and other monocots, but rarely in eudicots (Lan et al. 2025). Most aspects of tricin metabolism remain uncharacterized, whereas its biosynthetic pathway has been fully elucidated only in rice (Lam et al. 2014, 2015, 2019, 2021). Importantly, little is known about the transcriptional regulation of tricin metabolism and, hitherto, no transcription factor characterized as a tricin-related regulator has been reported. Here we report on the genome-wide characterization of R2R3 MYB genes in the model C4 grass *Setaria viridis* (green foxtail). This panicoid grass is phylogenetically close to important crops such as maize, sorghum, and sugarcane and has been proposed as a model species for the study of diverse biological processes in grasses, including C4 photosynthesis, plant domestication, and cell wall biology (Cesarino et al. 2020). As desirable attributes for an model species, *S. viridis* shows short life cycle, small stature, simple growth requirements, and a small, diploid and sequenced genome (Mamidi et al. 2020), as well as available stable genetic transformation protocols (Finley et al. 2021; Martins et al. 2015). First, we surveyed the *S. viridis* genome to identify all R2R3 MYB members and characterize them by means of in silico analyses, which included phylogenetic analysis and chromosome distribution. Subsequently, a combination of high-throughput expression analysis, co-expression analysis, RT-qPCR analysis, and transactivation assays led to the identification of three novel potential regulators of lignin/tricin metabolism in this model grass.

## Material and methods

### Identification and in silico characterization of *S. viridis* R2R3 MYB genes

To identify R2R3 MYB genes, we first performed "keyword search" against the *S. viridis* genome version 4.1 (Phytozome) using the PFAM domain PF00249, which is characteristic of MYB proteins (Shelton et al. 2012), yielding 267 sequences. These protein sequences were further evaluated for the presence of two MYB domains in the N-terminal of their corresponding polypeptides using both the NCBI Conserved Domain Search (https://www.ncbi.nlm.nih.gov/Structure/bwrpsb/bwrpsb.cgi) and SMART tool (Simple Modular Architecture Research Tool, http://smart.embl-heidelberg.de). Only proteins with typical R2R3 domain architecture were maintained, yielding 132 peptide sequences. Only the primary transcript for each gene was used, avoiding potential alternative splicing isoforms. Information regarding the physical distribution of *SvMYB*s across the 9 chromosomes of *S. viridis* was retrieved from Phytozome and a graphical representation was produced using MapGene2chromosome web v2 (http://mg2c.iask.in/mg2c_v2.0/). *SvMYB* genes falling within 50 kb of one another were considered as arising from tandem duplication (Cannon et al. 2004).

### Phylogenetic analysis

The complete MYB protein families from *Arabidopsis thaliana*, *Populus trichocarpa*, *Oryza sativa*, and *Brachypodium distachyon* were obtained from previous publications (Handakumbura et al. 2018; Soler et al. 2015). All protein sequences were aligned using the online version of MAFFT (https://mafft.cbrc.jp/alignment/server/index.html) and the resulting alignment was trimmed using trimAl (Capella-Gutiérrez et al. 2009) (http://phylemon2.bioinfo.cipf.es/index.html) with user define methods: minimum percentage of positions to conserve set to 10, gap threshold set to 0.5, similar threshold set to 0.0 and window size set to 1.0. The trimmed alignment was used for phylogenetic analysis using the Maximum Likelihood method and the online version of IQTree (http://iqtree.cibiv.univie.ac.at), with the best-fit substitution model automatically determined. Branch support analysis was performed from 1000 replicates applying the ultrafast bootstrap tests. The resulting ML tree was first generated with iTOL (Letunic and Bork 2024) (https://itol.embl.de) and then formatted as publication-ready figure using Graphic for Mac. Trees was drawn to scale and branch lengths represent the number of amino acid substitutions per site. Classification of the different R2R3 MYB subgroups was performed as previously reported for Arabidopsis (Dubos et al. 2010) and poplar (Soler et al. 2015). Given that the seminal classification of R2R3 MYBs based on Arabidopsis MYB proteins phylogeny (Dubos et al. 2010) results in subgroups S1 to S25, additional groups were not classified as subsequent numbers to avoid confusion with other reported studies. We further followed the classification described by Soler and colleagues (Soler et al. 2015) for the additional groups found in common with their study and named other groups according to the presence of SvMYB members.

### In silico gene expression and co-expression analyses

Gene expression data of *SvMYB* genes in various *S. viridis* organs and growth conditions were obtained from Phytozome Biomart tool (https://phytozome-next.jgi.doe.gov/biomart), using the “*S. viridis* v2.1 GeneAtlas v2 TPM” dataset. After compiling the expression values of *SvMYBs* in an Excel sheet, clustering and heatmap generation was performed using the web-based tool ClustVis (https://biit.cs.ut.ee/clustvis/). Evaluation of the expression profiles of *SvMYBs* in the elongating internode of *S. viridis* was carried out exploring a previously published dataset (Martin et al. 2016). RNAseq analysis was performed for the four functional zones of the elongating internode: meristematic zone (MsZ), zone of cell expansion (CEZ), transitional zone (TZ), and mature zone (MZ). The absolute transcript abundance for all *SvMYBs*, calculated as fragments per kilobase per million (FPKM), was obtained from the Additional File 2 of the mentioned study (Martin et al. 2016). Clustering and heatmap generation were also performed using the web-based tool ClustVis.

Pearson correlated expression data were retrieved for each *SvMYB* gene from PhytoMine (https://phytozome.jgi.doe.gov/phytomine/begin.do). After keyword search using the accession number of each *SvMYB* gene, a list of co-expressed genes with Pearson correlation values above 0.85 (the minimum threshold adopted by PhytoMine) was downloaded from the platform and the identification of genes putatively involved in different target functional categories was performed manually as described (Lima et al. 2023).

### Plant material and expression analysis via RT-qPCR

Plants of *S. viridis* var. A10 were grown on a 2:1 mixture of substrate enriched with macro- and micronutrients (Tropstrato HT, Vida Verde) and vermiculite upon 16/8 h light/dark at 26 °C and 65% humidity. Young leaves and young roots were harvested seven days after seed germination, whereas the bottom and top of the fifth internode were harvested from plants at “half-head emergence” (Martin et al. 2016). For each organ, samples from several plants were pooled into four biological replicates. Plant material was ground in liquid nitrogen and used for total RNA extraction using ReliaPrep^™^ RNA Tissue Miniprep System (Promega). A total of 1 μg of RNA previously treated with RQ1 DNAse (Promega) was used as template for cDNA synthesis using iScriptTM cDNA Synthesis Kit (Bio-Rad), according to manufacturer’s instructions. Specific primers were designed to amplify a fragment within the 3′UTR region of each selected *SvMYB* gene using the PrimerQuest online tool (https://www.idtdna.com/Prime rQuest/Home/Index). Housekeeping genes *CULLIN* (CUL) and *ELONGATION FACTOR 1-ALPHA* (EF1α) were used as references for normalization (Martins et al. 2016). RT-qPCR experiments were performed as previously described (Ferreira et al. 2016) using GoTaq^®^ qPCR Master Mix (Promega) and QuantStudio 6 Flex Real-Time PCR System (Applied Biosystems^™^). Calculations were carried out as previously described (Ferreira et al. 2019).). Expression data were analyzed by One-Way ANOVA followed by Tukey’s post hoc test (*P* < 0.05). Primers used in this study can be found in Table S1.

### Protoplast transactivation assays

Cloning of the promoter regions of the lignin biosynthetic genes *Sv4CL1* (Sevir.1G065000; 1990 bp upstream of the open reading frame—ORF) and *SvCOMT1* (Sevir.6G053300; 2029 bp upstream of the ORF) was previously reported (Lima et al. 2023). A region 483 bp upstream of the *SvCYP75B4* (Sevir.9G244501) ORF was PCR-amplified from *S. viridis* genomic DNA and cloned into pDONR221 via BP Clonase (ThermoFisher). After confirming sequence identity via Sanger sequencing, the *SvCYP75B4* promoter was subcloned into the destination vector pGWL7 (https://gateway.psb.ugent.be) via LR Clonase (ThermoFisher) to generate the reporter vector driving the expression of the firefly luciferase gene. The coding sequences of *SvMYB18*, *SvMYB24*, and *SvMYB74*, flanked by the Gateway^™^ cloning sites attL1 and attL2 into pUC-Kan were ordered as entry clones from GenScript (USA). After confirmation of sequence identity by Sanger sequencing, all *SvMYB* ORFs were subcloned into the destination vector p2GW7 (https://gateway.psb.ugent.be) to generate the effector vectors, in which the constitutive *CaMV35S* promoter drives the expression of the target transcription factors. A normalization vector was used, in which the *CaMV35S* promoter drives the expression of the Renilla luciferase. As a negative control, the transcription factor was replaced by the coding sequence of the ß-glucuronidase (GUS) gene subcloned into p2GW7. Transactivation assays were performed in tobacco BY-2 protoplasts as previously described (Vargas et al. 2016). The activity of the firefly luciferase was normalized by the activity of the Renilla luciferase for each replicate. The assays were performed with six biological replicates, each containing 100 μL of protoplast solution (around 500 protoplasts per μL).

## Results

### The *S. viridis* genome harbors 132 *R2R3-SvMYB* genes widely distributed into previously reported phylogenetic subgroups

We surveyed 29,807 loci of *S. viridis* (Phytozome, *Setaria viridis* annotation v4.1) to identify genes encoding MYBs by searching for the Myb-like DNA-binding domain (PF00249). The resulting hits were assessed for the presence of two MYB domains in the N-terminal of their corresponding polypeptides and only proteins with typical R2R3 domain architecture were maintained. This approach identified 132 nonredundant R2R3-MYB genes/proteins, named *SvMYB1* to *SvMYB132* according to their distribution along the nine chromosomes. This number of family members is slightly higher than the 114 R2R3-MYBs reported for *S. italica* (Muthamilarasan et al. 2014) and the 106 members found in rice (Yanhui et al. 2006), similar to the 126 members of Arabidopsis (Yanhui et al. 2006) and smaller than the 180 R2R3 MYBs reported for poplar (Soler et al. 2015). *SvMYB* genes were unevenly distributed along the nine chromosomes of *S. viridis*, with chromosome 5 showing the highest number of genes (n = 24) and chromosome 8 showing the lowest (n = 6, Fig. S1). Additionally, we found that 22 *SvMYB*s have arisen from tandem duplication events (Fig. S1), which represent 16.6% of the *SvMYB* genes.

**Fig. 1:**
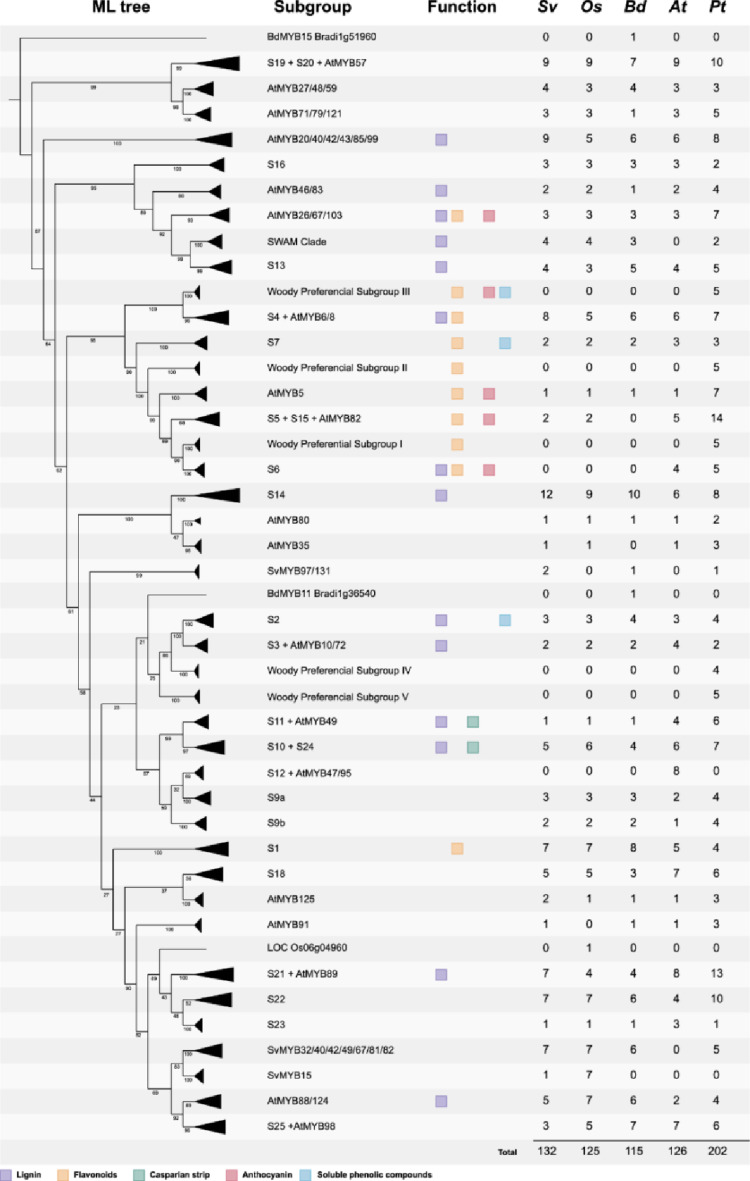
Maximum likelihood phylogenetic analysis of R2R3-MYB proteins from *Setaria viridis* (Sv), *Oryza sativa* (Os), Brachypodium distachyon (Bd), *Arabidopsis thaliana* (At), and *Populus trichocarpa* (Pt). Subgroups are collapsed and represented by black triangles. Bootstrap values are shown next to the branches. Subgroup names are included next to each clade. Subgroups with members previously associated with the biosynthesis of lignin (including in the context of Casparian strip formation), flavonoid, anthocyanin or soluble phenylpropanoids are identified with colored squares. The number of genes of each species for each subgroup is also included

To further assess the evolutionary history of *SvMYB*s genes, their corresponding protein sequences were aligned with those of Arabidopsis, poplar, rice, and *Brachypodium distachyo*n (Handakumbura et al. 2018; Soler et al. 2015) and their phylogenetic relationships were inferred by generating a maximum likelihood tree. Based on the topology of the resulting tree (Figs. 1, S2), we defined 43 subgroups that were named according to the following criteria: (i) if the subgroup contained at least one Arabidopsis MYB gene/protein, we used the nomenclature S1 to S25 established for Arabidopsis (Dubos et al. 2010; Stracke et al. 2001); (ii) for subgroups containing Arabidopsis MYBs falling outside the S1 to S25 grouping, we named the subgroup after the best-known functionally characterized Arabidopsis members, as previously reported (Soler et al. 2015); (iii) according to a previous work (Soler et al. 2015), groups containing only poplar MYBs were named “Woody Preferential Subgroups” and might represent genes involved in regulating aspects of cambium-derived woody growth; (iv) the SWAM clade was named following a previous publication (Handakumbura et al. 2018); and (v) to avoid confusion with previous publications reporting subgroups with numbers above S25 (Liu et al. 2017; Stracke et al. 2014), all the other subgroups were named according to the presence of SvMYB members. From the 43 subgroups, 27 (63%) contained members from all five species, whereas 16 were found only in some species (Figs. 1, S2). As expected, MYBs forming the different “Woody Preferential Subgroups” were found only in poplar, which was the only woody species included in our analysis. As previously reported, S12 was formed exclusively by Arabidopsis MYBs, which are known regulators of the Brassicaceae-specific glucosinolate biosynthesis (Matus et al. 2008). Interestingly, both “AtMYB35” and S5 + S15 + AtMYB82 were present in all species but Brachypodium. Whereas the former is a small subgroup with single or few members for each species, the latter clearly contained a higher number of members from eudicot species, especially poplar. Additionally, no grass MYBs were observed in group S6, which contained 4 Arabidopsis and 5 poplar members. Indeed, S5 and S6 were previously classified as putative “woody-expanded subgroups”, given their expansion in woody species including *E. grandis*, poplar and *Vitis vinifera* (Soler et al. 2015). Grouping of the SWAM clade was strongly supported and contained members from all species, except Arabidopsis, in agreement with previous observations suggesting that this clade is present in diverse angiosperms but absent from the Brassicaceae family (Handakumbura et al. 2018). Finally, 3 subgroups lacked Arabidopsis members and were not classified as Woody Preferential Subgroups. The subgroup “SvMYB15” was formed by seven MYBs from rice and a single SvMYB member. The subgroup “SvMYB97/131” was formed by two SvMYBs, one MYB from Brachypodium and one from poplar. The subgroup “SvMYB32/40/42/49/67/81/82” contained several members from all plant species, comprising a total of 25 MYBs.

Given our focus on the phenylpropanoid and lignin metabolism, we further included in our phylogenetic tree the information regarding the function of MYB transcription factors in regulating distinct branches of the phenylpropanoid pathway, including anthocyanins, flavonoids, soluble phenylpropanoids, and lignin (Fig. 1). We also indicated subgroups containing MYBs involved in the formation of Casparian strips, which are lignin-based cell wall structures serving as an apoplastic diffusion barrier in the root endodermis (Barbosa et al. 2019). However, although orthologous relationships allow functional inference and, thus, the identification of SvMYBs potentially related to the phenylpropanoid/lignin metabolism in *S. viridis*, using this type of information is limited by the conspicuous phylogenetic distance among target species. Comparing eudicots and grasses often leads to one-to-multiple orthologous relationships, precluding the efficient identification of genes with similar functions. Moreover, gene duplication is known to promote functional diversification (Wang et al. 2012), further complicating the selection of candidates. Therefore, here we adopted a pipeline based on multiple transcriptomic and co-expression analyses to identify SvMYBs potentially involved in lignification in *S. viridis*.

### Five SvMYBs were identified as potential regulators of lignin deposition in *S. viridis*

**Fig. 2:**
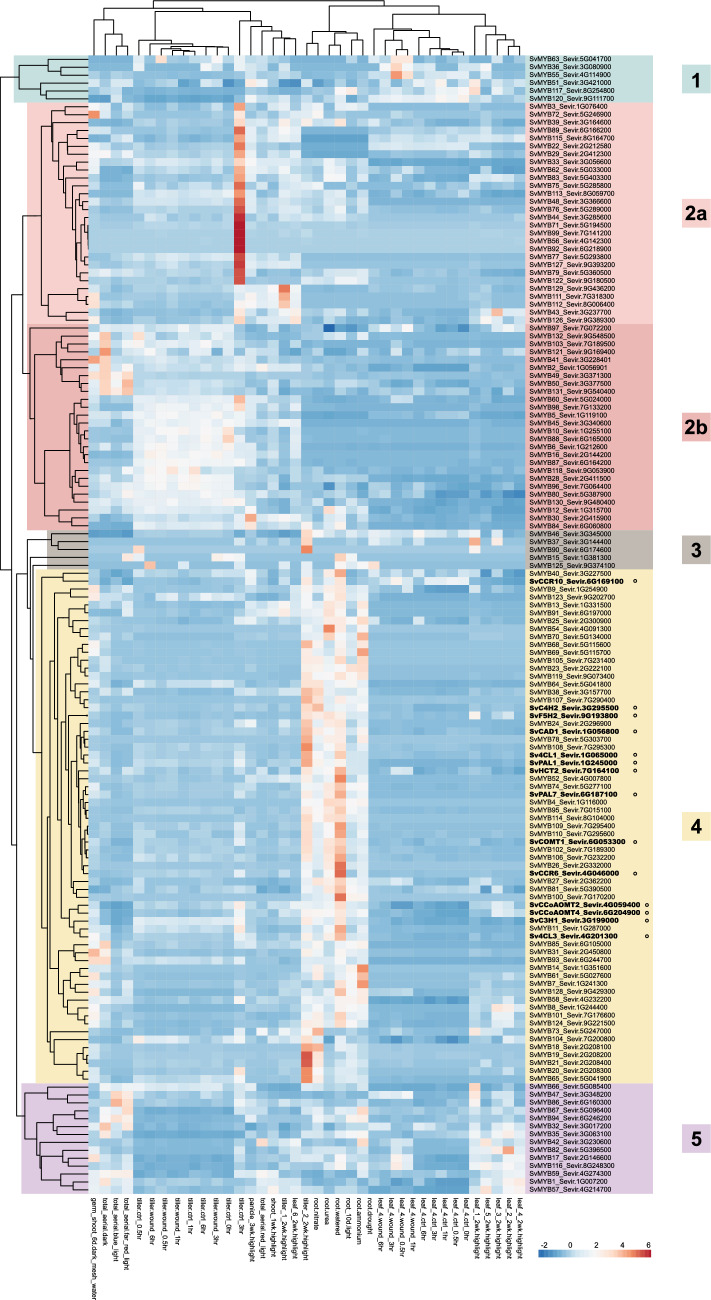
Expression profile of 132 *R2R3-SvMYB*s and the 14 genes identified as the lignin toolbox in *S. viridis* (highlighted in bold and marked with a circle) across 39 different organs and/or growth conditions. Transcript abundance is expressed in TPM values normalized using unit variance scaling, which is applied to rows. The color scale represents expression values, with blue indicating low and red indicating high expression. Clusters of co-expressed genes (1–5) are highlighted with different colors. *4CL* 4-coumarate:CoA ligase; *C3′H* *p*-coumarate 3-hydroxylase; *C4H* cinnamate 4-hydroxylase; *CAD* cinnamyl alcohol dehydrogenase; *CCoAOMT* caffeoyl-CoA *O*-methyltransferase; *CCR* cinnamoyl-CoA reductase; *COMT* caffeic acid *O*-methyltransferase; *F5H* ferulate 5-hydroxylase; *HCT* *p*-hydroxycinnamoyl-CoA:quinate/shikimate *p*-hydroxycinnamoyltransferase; *PAL* phenylalanine ammonia-lyase

The approach to identify candidate *SvMYB*s followed a similar pipeline used for other lignin-related genes in *S. viridis* (Cunha et al. 2025; Ferreira et al. 2019; Lima et al. 2023; Simões et al. 2023, 2020a; Sung et al. 2021), which was based on multiple in silico expression analyses, co-expression analysis and RT-qPCR. As a first step, we analyzed the expression pattern of *SvMYB*s using the “Gene Atlas Tissue Sample” from the Phytomine platform, which presents transcript abundance for a variety of tissue types, developmental stages, and abiotic stimuli in *S. viridis*. Despite the breadth of this set, none of the sample types represents a reference sample for active lignification. To overcome this limitation, we also included the 14 genes previously defined as the “lignin toolbox” in *S. viridis*, which corresponds to the core set of lignin biosynthetic genes (Ferreira et al. 2019), and organized the data as a heatmap to group genes with similar expression patterns. *SvMYB*s showing a similar expression pattern and, thus, clustering together with lignin biosynthetic genes in this broad set of *S. viridis* organs/conditions were considered as good candidates. The analysis depicted five major clusters of co-expressed genes (Fig. 2). Cluster 1 was formed by genes preferentially expressed in different leaf samples. Cluster 2 contained genes preferentially expressed in different tissues from the aerial part of the plant. Cluster 2 was further divided into group 2a, whose genes are more highly expressed in panicles, and group 2b, whose genes are more highly expressed in tillers. Cluster 3 contained only a few genes with broad expression patterns, without any obvious tissue specificity. Cluster 4 was the largest group, containing genes with preferential expression in roots. Finally, cluster 5 comprised genes with high expression in both leaves and total aerial parts of the plant. All lignin biosynthetic genes clustered in cluster 4, together with 51 *SvMYB* genes (Fig. 2). Based on their co-expression with genes encoding the enzymes responsible for the biosynthesis of monolignols, these 51 *SvMYB*s were initially selected as top candidates to play a role in the regulation of lignification in *S. viridis*.

**Fig. 3:**
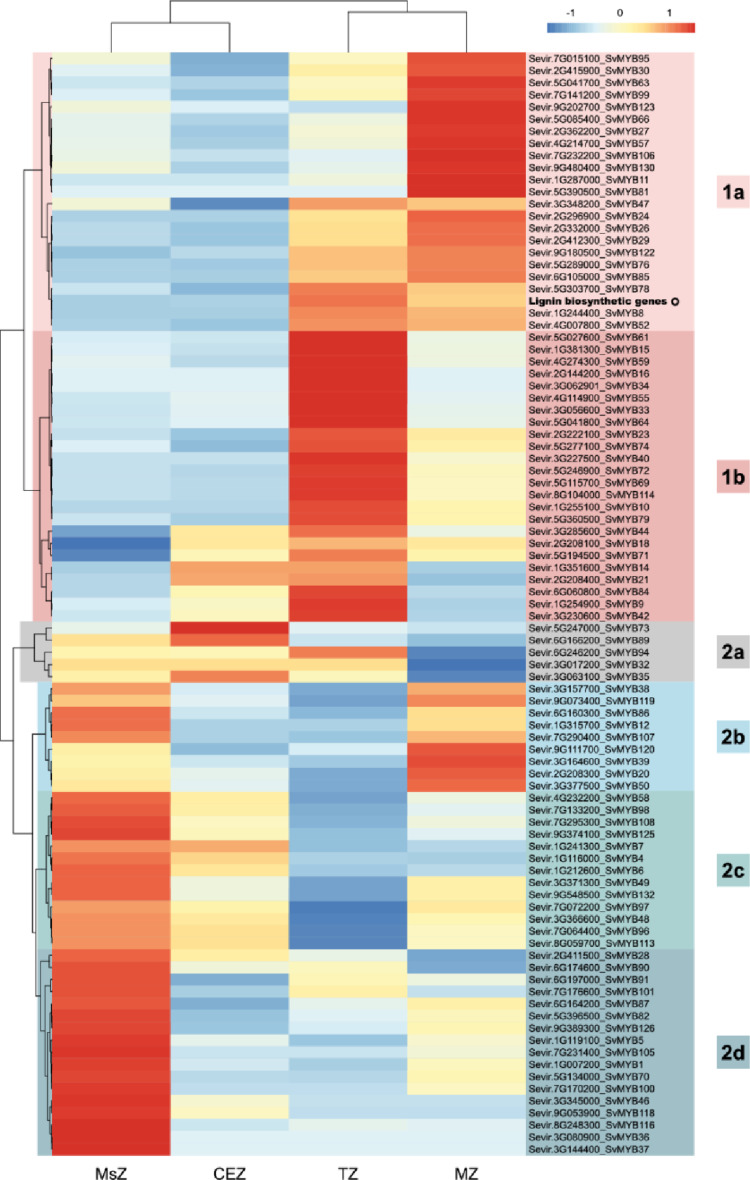
Expression profile of *R2R3-SvMYB*s in the four developmental zones of the *S. viridis* elongating internode. Expression data were retrieved from Martin et al. (2016). An artificial terminal with the mean expression profile of lignin biosynthetic genes (bold) was included to identify *SvMYB*s with similar patterns. Transcript abundance is expressed in FPKM values normalized using unit variance scaling. Clusters of co-expressed genes are highlighted with different colors. *MsZ* meristematic zone; *CEZ* cell expansion zone; *TZ* transitional zone; *MZ* maturation zone

To narrow down our list of candidate genes, we further evaluated the expression of *SvMYB*s along the elongating internode of *S. viridis*. In grasses, developing internodes contain four functional zones, from bottom to top: (i) a meristematic zone (MsZ), in which cells are actively dividing; (ii) a cell elongation zone (CEZ), formed by elongating cells; (iii) a transition zone (TZ), in which cell elongation no longer occurs and SCW deposition begins; and (iv) a maturation zone (MZ), in which sugar storage occurs concomitantly with SCW deposition (Ferreira et al. 2022; Martin et al. 2016; Zhang et al. 2014). Consequently, a gradient of SCW deposition is observed along the internode, with the content of all three components of SCW increasing from bottom to top. Large-scale transcriptomic analysis of the *S. viridis* elongating internode revealed that the transcript levels of phenylpropanoid-related genes (including both lignin and flavonoids) are low in MsZ and CEZ but strongly increases in TZ and MZ (Martin et al. 2016). Thus, *SvMYB*s showing an expression pattern similar to that observed for lignin genes in the elongating internode of *S. viridis* were considered good candidates. From the 132 *SvMYB*s found in the genome of *S. viridis*, expression data for 90 genes were available in the dataset (Table S2), whereas the remaining genes might not be expressed in culms. Expression data from *SvMYB*s were organized in a heatmap, which also included the representative expression pattern of lignin biosynthetic genes for reference (Fig. 3). The analysis depicted two major clusters: whereas cluster 1 contained genes with higher expression in TZ and/or MZ, cluster 2 comprised genes preferentially expressed in MsZ and/or CZ. These clusters were further subdivided to depict a more refined expression pattern. Cluster 1a included genes whose expression increased in TZ but peaked at MZ and cluster 1b included genes with their highest expression at TZ (Fig. 3). Cluster 2 was subdivided into four subclusters: cluster 2a contained genes with higher expression in both MsZ and CZ but peaking at CZ; cluster 2b included genes with higher expression in both MsZ and MZ; cluster 2c contained genes with higher expression in both MsZ and CZ but peaking at MsZ; and cluster 2d comprised genes preferentially expressed in MsZ. A total of 46 *SvMYB*s found in both clusters 1a and 1b showed an expression pattern compatible with a function in phenylpropanoid/lignin metabolism. Considering both in silico transcriptomic analyses, 22 genes showed a similar expression pattern to that of lignin biosynthetic genes i) in the “Gene Atlas Tissue Sample” of *S. viridis* tissues, and ii) in the elongating internode of *S. viridis*, and thus, were used for further analyses.

Co-expression analyses have been extensively employed to identify genes involved in different aspects of SCW deposition, given that the expression of SCW biosynthetic genes is highly coordinated both spatially and temporally (Cesarino et al. 2013; Ferreira et al. 2022; Simões et al. 2020b; Vanholme et al. 2013). Considering the principle of “guilt-by-association”, transcriptionally coordinated genes tend to function in the same biological process (Delli-Ponti et al. 2021; Ruprecht and Persson 2012). Therefore, genes with unknown function that are co-expressed with lignin-related genes might also play a role in lignin metabolism. Using this rationale, we further identified *SvMYB*s that were co-expressed with phenylpropanoid/lignin-related genes in a co-expression dataset available at the Phytomine platform, which is based on the same “Gene Atlas Tissue Sample” expression dataset. Given that MYBs are known to regulate different branches of the phenylpropanoid pathway, target co-expressed genes were classified into three categories: (i) phenylpropanoid-related (e.g., flavonoid and phenolic acid biosynthetic genes); (ii) lignin biosynthetic genes; and (iii) lignin-related (e.g., phenoloxidases, lignin-related transcription factors). Among the 22 candidate *SvMYB*s, 16 showed none or very few (n < 8 genes) co-expressed genes belonging to our target categories (Table S3). The remaining 6 *SvMYB*s were co-expressed with at least 11 target genes and included *SvMYB18* (n = 17 genes), *SvMYB23* (n = 17 genes), *SvMYB24* (n = 27 genes), *SvMYB74* (n = 32 genes), SvMYB95 (n = 11 genes), and *SvMYB106* (n = 27 genes). *SvMYB24* and *SvMYB74* were considered top candidates because their lists of co-expressed genes were enriched with (i) genes previously characterized as part of the lignin toolbox in *S. viridis* (Ferreira et al. 2019), (ii) genes encoding laccases previously selected as lignin-related in *S. viridis* (Simões et al. 2020a), and (iii) class III peroxidases previously characterized as lignin-related in *S. viridis* (Simões et al. 2023) (Table 1). Additionally, some of the co-expressed genes were homologues of master regulators of SCW deposition from the NAC family (Hussey et al. 2011; Zhong et al. 2021) or of hydroxycinnamaldehyde desidrogenases involved in the biosynthesis of wall-bound hydroxycinnamates (Nair et al. 2004; Yamamoto et al. 2024) (Table 1). Altogether, these results suggest that *SvMYB18*, *SvMYB23*, *SvMYB24*, *SvMYB74*, SvMYB95, and *SvMYB106* were good candidates to be involved in the regulation of lignification in *S. viridis*, with *SvMYB24* and *SvMYB74* as the most promising.

**Table 1:** List of phenylpropanoid- and lignin-related genes co-expressed with selected *SvMYB24* and *SvMYB74*

SvMYB	Correlated genes	Annotation	Category	Note	Correlation
SvMYB24 Sevir.2G296900	Sevir.5G268700	NAC DOMAIN CONTAINING PROTEIN 73	Lignin-related	Related to AtSND2/AtNAC73	0.973
	Sevir.J014600	Flavonoid 3′-monooxygenase/flavonoid 3′-hydroxylase	Phenylpropanoid-related		0.958
	Sevir.9G244500	Flavonoid 3′-monooxygenase/flavonoid 3′-hydroxylase	Phenylpropanoid-related		0.954
	Sevir.5G371400	Laccase	Lignin-related	Related to AtLAC5 and AtLAC12	0.935
	Sevir.2G228900	Flavonoid 3′-monooxygenase/Flavonoid 3′-hydroxylase	Phenylpropanoid-related		0.925
	Sevir.4G252600	NAC DOMAIN CONTAINING PROTEIN 75-related	Lignin-related	Related to AtSND4/AtNAC75	0.911
	Sevir.5G382900	Laccase	Lignin-related	Related to AtLAC17	0.907
	Sevir.8G224900	Laccase	Lignin-related		0.894
	Sevir.8G221900	Laccase	Lignin-related		0.893
	Sevir.6G231400	Class III peroxidase	Lignin-related		0.890
	Sevir.5G216900	Hydroxycinnamaldehyde dehydrogenase (ALDH2C/REF1)	Lignin-related	Related to HCALDH	0.888
	Sevir.7G269000	Shikimate kinase	Phenylpropanoid-related		0.887
	Sevir.6G187100	Phenylalanine/tyrosine ammonia-lyase (PTAL)	Lignin biosynthesis	Part of the lignin toolbox in *S. viridis*	0.886
	Sevir.4G195000	Class III peroxidase	Lignin-related		0.885
	Sevir.5G383000	Laccase	Lignin-related	Related to AtLAC17	0.883
	Sevir.9G228100	Feruloyl esterase/hydroxycinnamoyl esterase	Phenylpropanoid-related		0.882
	Sevir.3G277100	Class III peroxidase	Lignin-related		0.881
	Sevir.8G262800	Laccase	Lignin-related	Related to AtLAC4	0.879
	Sevir.7G178200	Phenylalanine/tyrosine ammonia-lyase (PTAL)	Lignin biosynthesis	Part of the lignin toolbox in *S. viridis*	0.875
	Sevir.4G044900	Hydroxycinnamoyl CoA:shikimate hydroxycinnamoyl transferase (HCT)	Lignin biosynthesis		0.873
	Sevir.2G339400	Laccase	Lignin-related		0.864
	Sevir.1G056800	Cinnamyl alcohol dehydrogenase (CAD)	Lignin biosynthesis	Part of the lignin toolbox in *S. viridis*	0.862
	Sevir.9G395800	Flavonoid 3′,5′-hydroxylase (CYP75A)	Phenylpropanoid-related		0.860
	Sevir.5G383300	Laccase	Lignin-related	Related to AtLAC17	0.859
	Sevir.3G412600	( +)-pinoresinol reductase/lariciresinol reductase	Lignin-related		0.857
	Sevir.8G016500	Laccase	Lignin-related	Related to AtLAC5 and AtLAC12	0.853
SvMYB74 Sevir.5G277100	Sevir.6G187100	Phenylalanine/tyrosine ammonia-lyase (PTAL)	Lignin biosynthesis	Part of the lignin toolbox in *S. viridis*	0.945
	Sevir.9G395800	Flavonoid 3′,5′-hydroxylase (CYP75A)	Phenylpropanoid-related		0.945
	Sevir.5G216900	Hydroxycinnamaldehyde dehydrogenase (ALDH2C/REF1)	Lignin-related	Related to HCALDH	0.931
	Sevir.8G222400	Laccase	Lignin-related		0.922
	Sevir.5G383300	Laccase	Lignin-related	Related to AtLAC17	0.920
	Sevir.1G245100	Phenylalanine ammonia-lyase (PAL)	Lignin biosynthesis		0.917
	Sevir.6G231400	Class III peroxidase	Lignin-related		0.914
	Sevir.7G178200	Phenylalanine/tyrosine ammonia-lyase (PTAL)	Lignin biosynthesis	Part of the lignin toolbox in *S. viridis*	0.913
	Sevir.8G221600	Laccase	Lignin-related		0.906
	Sevir.2G143400	Flavone/flavonol 7-*O* -beta-d-glucoside malonyltransferase	Phenylpropanoid-related		0.903
	Sevir.7G337400	Class III peroxidase	Lignin-related	Lignin-related according to Simões et al (2023)	0.903
	Sevir.4G044900	Hydroxycinnamoyl CoA:shikimate hydroxycinnamoyl transferase (HCT)	Lignin biosynthesis	Part of the lignin toolbox in *S. viridis*	0.902
	Sevir.5G268700	NAC DOMAIN CONTAINING PROTEIN 73	Lignin-related	Related to AtSND2/AtNAC73	0.902
	Sevir.2G003200	Laccase	Lignin-related		0.900
	Sevir.8G262800	Laccase	Lignin-related	Related to AtLAC4	0.896
	Sevir.4G201300	4-Coumarate:CoA ligase (4CL)	Lignin biosynthesis	Part of the lignin toolbox in *S. viridis*	0.892
	Sevir.3G223200	Laccase	Lignin-related	Related to AtLAC17	0.885
	Sevir.6G244700	MYB42/85	Lignin-related	Related to AtMYB42/85	0.884
	Sevir.4G195000	Class III peroxidase	Lignin-related		0.881
	Sevir.8G013900	Class III peroxidase	Lignin-related		0.880
	Sevir.1G245000	Phenylalanine/tyrosine ammonia-lyase (PTAL)	Lignin biosynthesis	Part of the lignin toolbox in *S. viridis*	0.879
	Sevir.9G368000	Flavonoid 3′-monooxygenase/flavonoid 3′-hydroxylase	Phenylpropanoid-related		0.877
	Sevir.3G277100	Class III peroxidase	Lignin-related		0.872
	Sevir.8G223000	Laccase	Lignin-related		0.871
	Sevir.1G016500	Benzyl alcohol *O* -benzoyltransferase	Phenylpropanoid-related		0.870
	Sevir.J014600	Flavonoid 3′-monooxygenase/flavonoid 3′-hydroxylase	Phenylpropanoid-related		0.867
	Sevir.2G296900	MYB42/85	Lignin-related	Related to AtMYB42/85	0.865
	Sevir.5G093900	*O* -succinylbenzoate synthase	Phenylpropanoid-related		0.864
	Sevir.4G287600	Benzyl alcohol *O*-benzoyltransferase	Phenylpropanoid-related		0.864
	Sevir.6G093500	4-Coumarate:CoA ligase (4CL)	Lignin biosynthesis		0.858
	Sevir.9G244500	Flavonoid 3′-monooxygenase/flavonoid 3′-hydroxylase	Phenylpropanoid-related		0.858
	Sevir.8G221000	Laccase	Lignin-related		0.857

Only genes with Pearson Correlation Score above 0.85 are shown

**Fig. 4:**
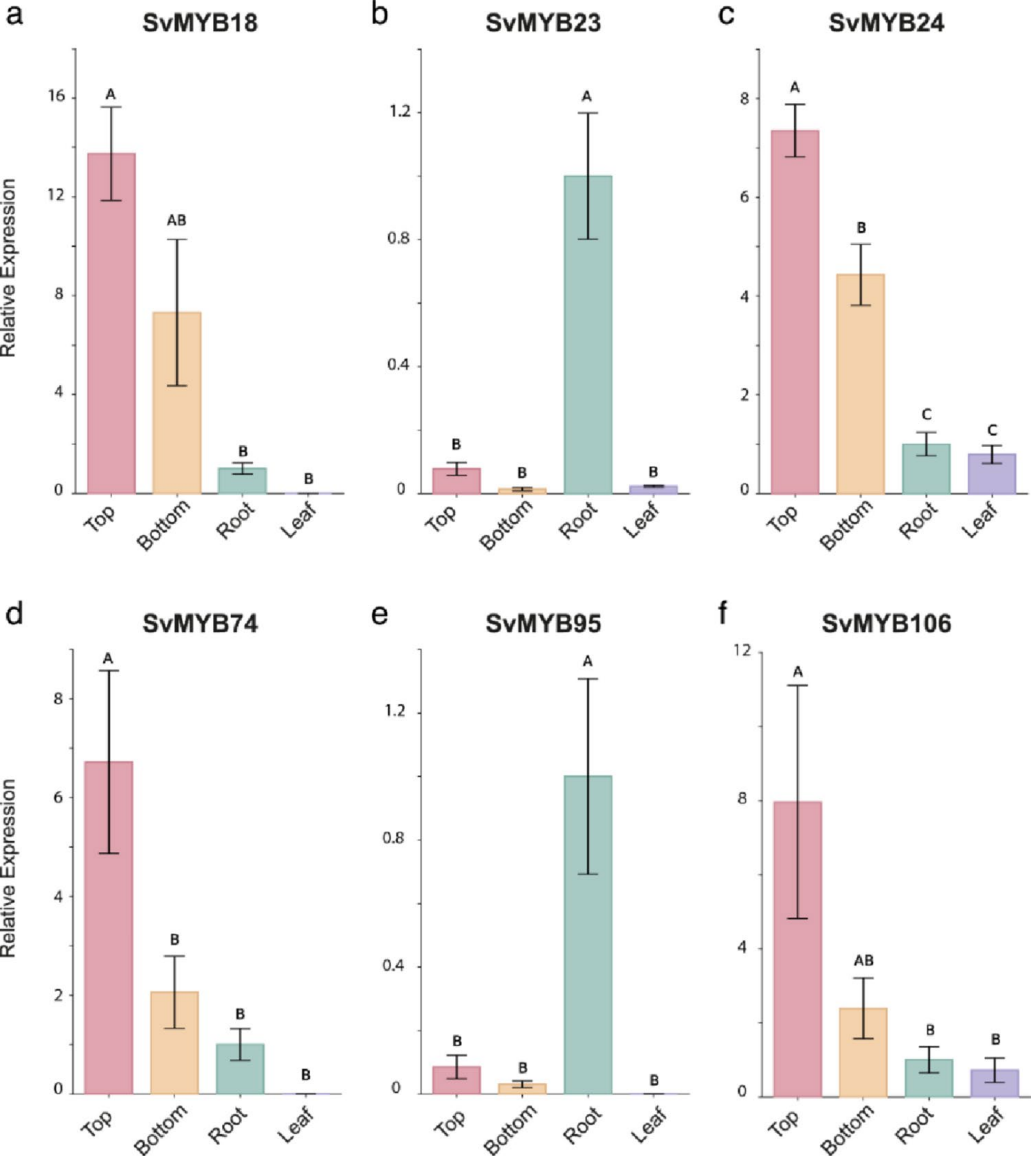
Expression of selected *SvMYB*s in tissues of *S. viridis* contrasting for lignin content determined by RT-qPCR. “Top” and “bottom” refer to the regions of the elongating internode*.* Error bars indicate SE. Differences were assessed with One-Way ANOVA and Tukey’s post hoc test (*P* < 0.05, n = 4)

As the last step in selecting lignin-related *SvMYB*s, we performed expression analysis to compare the transcript profile of candidate genes with the pattern of lignin deposition. Accordingly, RT-qPCR was carried out in four *S. viridis* tissues contrasting for their lignin content: young roots, young leaves, bottom and top of the elongating internode. *SvMYB*s with high expression in the top of the elongating internode, a tissue undergoing active lignification (Martin et al. 2016), were considered our best candidates. *SvMYB23* and *SvMYB95* showed strong and preferential expression in young roots, with very low expression levels in all other tissues (Fig. 4). The other four genes were highly and preferentially expressed in the top of the elongating internode (Fig. 4), the expression profile observed for members of the lignin toolbox and other lignin-related genes in *S. viridis* (Ferreira et al. 2019; Simões et al. 2020a). Thus, considering our complete pipeline for the identification of lignin-related genes, we concluded that *SvMYB18, SvMYB24, SvMYB74,* and *SvMYB106* are potentially involved in the regulation of lignin deposition in *S. viridis*.

### *SvMYB18, SvMYB24 *and* SvMYB74* activate the promoter of lignin and tricin biosynthetic genes

**Fig. 5:**
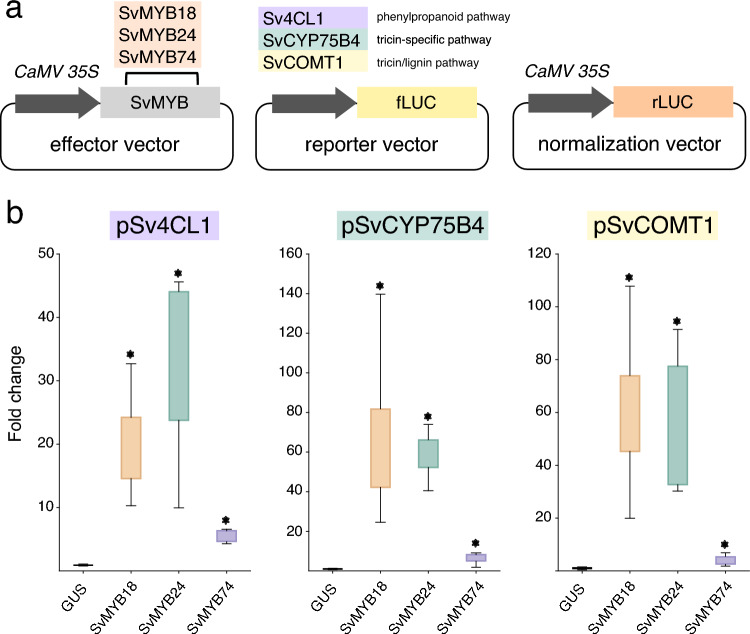
Transactivation analysis of lignin and tricin-related promoters by candidate SvMYBs in tobacco BY-2 protoplasts. **a** Schematic representation of the effector, reporter and normalization vectors used for transactivation assays. In the effector vector, the constitutive *CaMV 35S* promoter drives the expression of selected *SvMYB*s, while in the reporter vectors the promoters of lignin and tricin biosynthetic genes are individually fused with the firefly luciferase reporter gene (fLUC). The different promoters are highlighted with different colors. The normalization vector consisted of the *CaMV 35S* promoter driving the expression of the renilla luciferase (rLUC) reporter gene. **b** Transactivation analysis showing the activation of lignin and tricin biosynthetic genes by all three candidate SvMYBs. Values are fold-changes normalized to protoplasts co-transfected with the reporter construct and a *CaMV 35S::GUS* negative control plasmid. Error bars indicate the standard deviation and significance between each transcription factor and the negative control (GUS) was determined by Student’s t test (*P < 0.05; n = 6)

To gain further insights into the regulatory function of the selected SvMYBs in the context of lignification, we evaluated their ability to activate the expression of lignin biosynthetic genes using transactivation assays in tobacco BY-2 protoplasts. SvMYB106 was excluded from this analysis because we have previously reported its ability to activate the promoters of the lignin biosynthetic genes *Sv4CL1*, *SvCOMT1*, and *SvF5H2* (please note that the gene was back then referred to as SvMYB76) (Lima et al. 2023). In addition to contribute to the identification of regulators of lignin metabolism in *S. viridis*, we also aimed to gain insights into molecular mechanisms underlying grass-specific aspects of lignification. The flavone tricin was recently identified as an authentic lignin monomer in grass lignin (del Río et al. 2012; Lan et al. 2016, 2015), but little is known about molecular aspects of tricin metabolism. Importantly, although its biosynthetic pathway has been fully elucidated in rice (Lam et al. 2021), so far no transcription factor was characterized with the ability to specifically regulate tricin biosynthetic genes. Thus, for our screening with the transactivation assay, we selected the promoters of three key genes: (i) *Sv4CL1*, which was previously indicated as part of the lignin toolbox in *S. viridis* and encodes an enzyme working in the general phenylpropanoid pathway (Ferreira et al. 2019); (ii) *SvCYP75B4*, which encodes the only enzyme specifically involved in tricin biosynthesis (Lam et al. 2015); and (iii) *SvCOMT1*, a bifunctional *O*-methyltransferase working in the biosynthesis of sinapyl alcohol (giving rise to S units upon polymerization into the lignin polymer) and tricin (Eudes et al. 2017; Lam et al. 2019). These promoters were inserted upstream of the firefly luciferase gene to generate reporter plasmids, whereas the expression of candidate transcription factors was driven by the constitutive 35S promoter to generate effector plasmids (Fig. 5a). Both plasmids were transfected into tobacco BY-2 protoplasts and luciferase activity was measured to evaluate transcriptional activity. We found that all three SvMYBs activated the tested promoters, although with different activation strengths (Fig. 5b). Whereas both SvMYB18 and SvMYB24 showed strong transactivation activity, activating the target promoters by more than 20-fold when compared to the control, the activity of SvMYB74 was conspicuously lower. Within the limits of our experimental system, these results suggest that our pipeline was successful in identifying lignin-related SvMYBs and that our candidates regulate the expression of lignin and tricin biosynthetic genes in *S. viridis*.

## Discussion

R2R3-MYBs are regulators of distinct branches of the phenylpropanoid pathway, playing important roles in plant development and in their responses to environmental stimuli. In the context of lignification, R3R3-MYBs are involved in different levels of the complex hierarchical transcriptional network controlling SCW deposition. Upstream of the NAC-MYB conserved network, MYB26 control the cell wall thickening program via the direct induction of the top layer master regulators NST1 and NST2 (Yang et al. 2017). Within the network, MYB46/83 are master regulators of the second layer, controlling not only SCW biosynthetic genes but also activating downstream transcription factors from different gene families (McCarthy et al. 2009; Zhong et al. 2007). In this bottom layer of the network, several R2R3-MYBs from diverse subgroups play specific and non-redundant roles in lignin metabolism, controlling lignin content and composition. Although several R2R3-MYBs placed in the different layers of this network and from distinct species have been characterized with a function in lignification, there is still much to be explored in terms of regulation of specific aspects of grass lignin. For instance, misregulation of selected *MYB*s in grasses led to altered levels of lignin *p*-coumaroylation (Bhatia et al. 2019; Miyamoto et al. 2019; Scully et al. 2016; Shen et al. 2012), but very few of these studies demonstrated an effect on the expression of genes encoding *p*-coumaroil-CoA:monolignol transferases (PMTs) (Miyamoto et al. 2019; Zhao et al. 2019), the enzymes responsible for monolignol acylation with *p*-coumarate. Still, direct binding of these MYBs to the promoter of *PMT*s was not demonstrated, and the possibility that the activation of *PMT* expression may rely on interactions with other downstream transcription factors cannot be excluded. Thus, the distinct structural aspects of grass lignin advocates for a detailed search and characterization of transcription factors associated with the regulation of such traits, and the R2R3-MYB family is certainly a prominent source (Miyamoto et al. 2020). In addition, MYBs are excellent targets for bioengineering of plant biomass towards crop improvement in the context of the bioeconomy, as regulators of specific branches of the phenylpropanoid pathway might be misregulated to promote the accumulation of beneficial metabolites or to alter lignin deposition to reduce biomass recalcitrance for biorefineries.

We took advantage of the recently developed genetic and genomic tools for the model grass *S. viridis* to identify R2R3-MYB transcription factors potentially involved in the regulation of lignin metabolism in grasses. The gene family comprises 132 members, which is higher than the 114 genes reported for *S. italica* (Muthamilarasan et al. 2014), the domesticated relative of *S. viridis* (Hu et al. 2018). Given that gene duplication is a major driving force towards functional diversification (Wang et al. 2012), the conspicuous large size of the MYB family in *S. viridis* and other plant species suggests extensive events of sub- and neofunctionalization and highlight the importance of MYB transcription factors in plant growth and development and in the interaction of plants with their surrounding environments (Dong and Lin 2021; Zhang et al. 2025). Phylogenetic analysis showed that SvMYBs were broadly distributed among previously reported subgroups, several of them containing members whose function in regulating different branches of the phenylpropanoid pathway has been previously reported. We observed a good agreement between the topology of our phylogenetic tree and that previously described for *Eucalyptus grandis* by Soler et al (2015), with basically the same subgroups. The exceptions were (i) the following subgroups were merged: S5 + S15 + AtMYB82, S12 + AtMYB47/95, S19 + S20 + AtMYB57; (ii) the following subgroups clustered separated: S2 from S3 + AtMYB10/72, S13 from AtMYB26/67/103; and (iii) S18 was not separated into S18a and S18b (Figs. 1, S2). These differences may be a consequence of the different datasets used for the different phylogenies: whereas Soler et al (2015) sampled four eudicot woody species and one grass species, our phylogeny contained three grasses, one eudicot tree and one eudicot herbaceous plant. This fact frequently impaired the identification of orthologous relationships between MYBs from different species, given the phylogenetic distance between eudicots and grasses and, consequently, the occurrence of events of lineage-specific gene duplication and/or gene loss.

Members from several subgroups were demonstrated to be involved in the regulation of lignin deposition. Considering the evolutionarily conserved NAC-MYB regulatory network, members of the subgroup AtMYB46/83 comprise second-level master switches capable of activating the complete program of secondary wall deposition, acting upstream of other lignin-related regulators (McCarthy et al. 2009; Zhong et al. 2007; Zhong and Ye 2012). Within the third level of the network, which is formed by numerous transcription factors from several gene families, MYBs from different subgroups operate as direct regulators of lignin biosynthetic genes. For instance, subgroups S2, S3 + AtMYB10/72, S4 + AtMYB6/8, S6, S10 + S24, S11 + AtMYB49, S13, S14, S21 + AtMYB89, SWAM clade, AtMYB20/40/42/43/85/99, AtMYB26/67/103, and AtMYB88/124 all contained lignin regulators in multiple plant species (Bhargava et al. 2010; Chen et al. 2025; Chezem et al. 2017; Handakumbura et al. 2018; Jin 2000; Kamiya et al. 2015; Liu et al. 2024; Newman et al. 2004; Ohman et al. 2013; Xu et al. 2022; Zhong et al. 2008; Zhou et al. 2009). The paralogues MYB58/63 belonging to S3 + AtMYB10/72 were among the first characterized direct activators of lignin deposition in Arabidopsis (Zhou et al. 2009), and their corresponding orthologues in other species showed conserved functions (Cesarino et al. 2016). Several members of subgroups S10 + S24, S11 + AtMYB49, and S14 are regulators of lignin deposition in the context of Casparian strips formation, both in Arabidopsis and rice (Chen et al. 2025; Kamiya et al. 2015; Wang et al. 2022; Xu et al. 2022), being part of a complex transcriptional network that balances lignification and suberization in the endodermis. Another interesting example is the SWAM clade, which belongs to the subgroup S13 and lacks representation in Brassicaceae. SWAM1 was characterized as an activator of cellulose- and lignin-associated gene expression, controlling secondary wall deposition (Handakumbura et al. 2018). Finally, some of these downstream MYBs act as regulators of specific aspects of lignification, such as AtMYB103 that was shown to specifically control syringyl (S)-lignin biosynthesis (Ohman et al. 2013).

MYBs have also been extensively characterized as regulators of flavonoid biosynthesis. Subgroups S1, S4 + AtMYB6/8, S5 + S15 + AtMYB82, S6, S7, AtMYB5, AtMYB26/67/103, and Wood Preferential Subgroups I, II and III contained members functioning in the control of distinct branches of the flavonoid pathway (Fig. 1) (Baudry et al. 2004; James et al. 2017; Jiang et al. 2022; Shi et al. 2022; Soler et al. 2016; Stracke et al. 2007; Tohge et al. 2005; Wang et al. 2020; Xu et al. 2014; Yoshida et al. 2015). Several of them regulate anthocyanin biosynthesis, including PRODUCTION OF ANTHOCYANIN PIGMENT1 (PAP1) and its downstream targets AtMYB113/114 from subgroup S6 (Gonzalez et al. 2008), whereas MYBs from subgroup S7 specifically regulate the synthesis of flavonols (Stracke et al. 2007) in Arabidopsis. Members of Wood Preferential Subgroups II and III from poplar were also recently characterized as regulators of proanthocyanidin and anthocyanin synthesis and structure (James et al. 2017; Yoshida et al. 2015), although these studies have not used the corresponding nomenclature established for the subgroups by Soler et al (2015). Interestingly, members of subgroup S7 appear to have broader specificity, as the overexpression of AtMYB12 in tomato activated the chlorogenic acid biosynthetic pathway in addition to flavonol biosynthesis, leading to the accumulation of diverse polyphenols (Luo et al. 2008). Members of subgroups related to flavonoid biosynthesis were also shown to regulate soluble phenolics other than chlorogenic acids. For instance, poplar plants overexpressing *EgMYB88* from *Eucalyptus grandis*, belonging to the Wood Preferential Subgroup I, exhibited a substantial increase in the levels of the flavonoid catechin and of some salicinoid phenolic glycosides, which was accompanied by higher transcript levels of corresponding biosynthetic genes (Soler et al. 2016). Regulators of soluble phenolic compounds were also found within subgroup S2, as VvMYB14 and VvMYB15 were shown to regulate stilbene biosynthesis in *Vitis vinifera* (Höll et al. 2013). These regulators are interesting in the context of bioengineering, given that soluble phenylpropanoids such as chlorogenic acids, flavonoids and stilbenes have been extensively characterized for their beneficial effects on human health (Li et al. 2021; Rates and Cesarino 2023).

Although orthology/homology-based functional inference is considered an effective strategy to select candidate phenylpropanoid-related R2R3-MYBs, the diversity of members playing similar (but non-redundant) functions in controlling different branches of this pathway advocates for additional strategies to narrow down the selection. Here, by employing multiple transcriptomic and co-expression analyses, we selected four candidate *SvMYB*s, whose role in the regulation of lignin deposition in *S. viridis* is supported by several lines of evidence: (i) similar expression profiles to that observed for the lignin toolbox genes in different organs/conditions of the “Gene Atlas Tissue Sample” from *S. viridis*; (ii) similar expression patterns to that of lignin biosynthetic genes along the *S. viridis* elongating internode; (iii) co-expression with several genes encoding phenylpropanoid- and lignin-related genes in the Phytomine public transcriptomic database; (iv) high expression levels in the top of the *S. viridis* elongating internode, a tissue undergoing active lignification. Combined expression and co-expression analyses have been demonstrated as a powerful tool for the selection of candidate SCW- and phenylpropanoid-related MYBs in several species (Rao et al. 2019; Yang et al. 2021a, b; Zhao et al. 2019), supporting the effectiveness of our approach.

Although the transcriptomic-based strategy employed in this work has been successful in the identification of lignin-related genes in *S. viridis* (Cunha et al. 2025; Lima et al. 2023; Simões et al. 2023, 2020a; Sung et al. 2021), it is noteworthy that our selection was mainly based on stem/internode tissues as the most relevant lignification sites, but we cannot exclude the possibility that *SvMYB*s preferentially expressed in other organs are also involved in the regulation of lignin deposition. The criterium of stem-preferential expression to select lignin-related genes has been classically employed in studies using woody species (Carocha et al. 2015; Saleme et al. 2017; Shi et al. 2010), given their massive xylem formation and, consequently, their conspicuously high lignin levels in stems. However, grasses tend to show a more uniform pattern of lignin deposition across organs, with higher lignin levels in leaves and roots when compared to weedy eudicot species (Zhu and Barros 2025). Additionally, not only the cell walls of vascular tissues are reinforced with lignins in all plant organs, but also lignin deposition occurs in very specific cellular contexts to allow functional specialization (Pesquet et al. 2025). For instance, a lignin-based apoplastic barrier named “neck strip” was recently described in glandular trichomes of different plant species (Hao et al. 2024), working to avoid apoplastic leakage of important metabolites from glandular cells to stalk and basal cells. Interestingly, the establishment of the neck strip in cucumber glandular trichomes is regulated by CsMYB36 (Hao et al. 2024), a homologue to the master regulator of Casparian strip formation AtMYB36 in Arabidopsis roots (Kamiya et al. 2015), although different sets of downstream genes might be regulated in each case. Similarly, AtMYB26 mediates the regulatory mechanism of rapid endothecium lignification for timely anther dehiscence and successful pollen release during Arabidopsis reproduction (Xue et al. 2024). Therefore, although our strategy was certainly effective to identify candidate SvMYBs potentially involved in stem lignification in *S. viridis*, several other members of the R2R3-MYB family likely regulate lignin deposition in other organs and/or cellular structures.

The four candidates selected by our approach were *SvMYB18, SvMYB24, SvMYB74* and *SvMYB106*, but the latter was not used in our transactivation assay due to two reasons. First, SvMYB106 belongs to the subgroup S3 + AtMYB10/72 and is (co)orthologous to the pair of lignin-specific activators AtMYB58/63 in Arabidopsis (Zhou et al. 2009). The function of these MYBs as lignin activators is evolutionarily conserved and has been extensively demonstrated in different grass species (Hirano et al. 2013; Rao et al. 2019; Scully et al. 2016). Although some these findings indicate that MYB58/63s might play a unique role in grasses by also activating the biosynthesis of cell-wall polysaccharides (Noda et al. 2015; Rao et al. 2019; Scully et al. 2016), this function may vary among grass species (Miyamoto et al. 2020) and evaluating it here was beyond the scope of this study. Second, we have demonstrated the ability of SvMYB106 in activating the promoters of several lignin biosynthetic genes in a previous work (Lima et al. 2023). SvMYB106, as well as its paralogue SvMYB11, activated the promoters of *Sv4CL1*, *SvCOMT1* and *SvF5H2*, genes belonging to the lignin toolbox of *S. viridis* (Ferreira et al. 2019). Interestingly, our pipeline was able to select *SvMYB11* until the step of co-expression analysis using the Phytomine dataset, but this gene was excluded for the subsequent steps because of the low number of co-expressed genes from target categories. Nevertheless, knowing that *SvMYB11* is (co)orthologous to AtMYB58/63 and that it can activate lignin promoters in transactivation assays (Lima et al. 2023), we have performed RT-qPCR analysis in the same set using for our candidate *SvMYB*s and observed the expected expression pattern for a lignin-related gene (i.e., high and preferential expression in the top of the elongating internode) (Fig. S3). These findings suggest that, although our pipeline was indeed effective in selecting strong lignin-related candidates, many other SvMYBs might be involved in lignification.

More important than to simply identify regulators of lignin deposition in *S. viridis*, the major goal of this study was to gain insights into the regulatory role of MYBs in grass-specific aspects of lignification. Although tricin has been identified as an authentic lignin monomer in grasses more than a decade ago, information about the molecular mechanisms underlying tricin biosynthesis remains scarce. Particularly, specific regulators of tricin biosynthesis remain unknown, and the few reports demonstrating any effect of misregulation of candidate transcription factors on tricin content (and incorporation into lignin) did not focus specifically on their role in regulating tricin biosynthetic genes. For instance, loss-of-function of *OsMYB108*, a rice homolog of subgroup S4 repressor *AtMYB4* in Arabidopsis (Jin 2000), resulted in higher levels of lignin-incorporated tricin in rice, which was accompanied by the upregulation of some tricin biosynthetic genes (Miyamoto et al. 2019). Additionally, overexpression of subgroup S13 *AtMYB61* from Arabidopsis in rice increased lignin content mainly by enriching S and tricin units in the lignin polymers (Koshiba et al. 2017). These results suggest that distinct MYBs might be closely associated with the coordinated regulation of lignin content and composition, but experiments showing the capacity of candidate transcription factors in regulating tricin biosynthetic genes are still missing. Here, SvMYB18, SvMYB24 and SvMYB74 activated the promoter of the general phenylpropanoid gene *Sv4CL1* but also of the tricin biosynthetic genes *SvCYP75B4* and *SvCOMT1*. Whereas the former encodes the enzyme responsible for the 3′-hydroxylation of apigenin and the 5′-hydroxylation of chrysoeriol [thus, it was also named apigenin 3′-hydroxylase/chrysoeriol 5′-hydroxylase, A3′H/C5′H, (Lam et al. 2021, 2015)] in the tricin pathway, the latter encodes a bifunctional *O*-methyltransferase catalyzing the 5′-*O*-methylation of 5-hydroxyconiferaldehyde in S-lignin pathway and the 3′-*O*-methylation of luteolin and the 5′-*O*-methylation of selgin in the tricin pathway (Eudes et al. 2017; Lam et al. 2019). To the best of our knowledge, this is the first time that the transactivation activity of a transcription factor on tricin biosynthetic genes is demonstrated. However, it is important to mention that this experiment does not provide information of the direct binding of a given transcription factor to the target promoter, as the activity could result from the interaction with other transcription factors from the BY-2 protoplasts that were expressed downstream of the overexpressed regulator. Nevertheless, as the tricin pathway is absent in tobacco, it is likely that our candidate SvMYBs indeed directly regulate the target biosynthetic genes.

Both SvMYB18 and SvMYB24 belong to subgroup AtMYB20/40/42/43/85/99, but they are not phylogenetically close to the same Arabidopsis homologues. In our phylogeny, the AtMYB20/40/42/43/85/99 subgroup was clearly divided into 3 major clades: (i) one formed by AtMYB99 alone, (ii) a smaller clade containing AtMYB42 and AtMYB85, (iii) and a major clade containing AtMYB20, AtMYB40, and AtMYB43. SvMYB18 was found in the AtMYB20/40/43 clade, whereas SvMYB24 was associated with the AtMYB42/85 clade (Fig. S2). MYB genes from both clades have been linked with lignification in Arabidopsis. AtMYB85 can target the lignin biosynthetic genes *At4CL1* and *AtHCT* (Geng et al. 2020; Zhong et al. 2008) and its overexpression led to ectopic deposition of lignin in epidermal and cortical cells in stems (Zhong et al. 2008). The function of MYB42/85 in the control of lignification is likely conserved, as the overexpression of their orthologues in switchgrass and maize resulted in a similar phenotype of increased lignin content and elevated S/G ratio (Bhatia et al. 2019; Rao et al. 2019). The transcriptional activity of SvMYB24 shown in this work is in line with the conserved function of members of this clade in lignification, but here we further demonstrated a potential regulatory role in tricin biosynthesis. Similarly, *AtMYB20* and *AtMYB43*, together with *AtMYB42* and *AtMYB85*, were shown to be downstream targets of master regulators of the NAC family (AtNST1) and MYB family (AtMYB46) and to coordinately (and positively) regulate phenylalanine availability and lignin biosynthesis (Geng et al. 2020). Interestingly, MYBs belonging to the S4 subgroup, the repressors *AtMYB4/7/32*, were downregulated in the *myb20/42/43/85* quadruple mutant, resulting in higher expression of the gene encoding chalcone synthase (CHS) and, consequently, higher levels of flavonoids (Geng et al. 2020). Thus, the authors concluded that AtMYB20/42/43/85 are activators of lignin deposition but indirect repressors of flavonoid biosynthesis, whose activity occurs via repression of flavonoid repressors from the subgroup S4. Here, we showed that SvMYB18 not only activated the promoters of genes encoding biosynthetic enzymes of the lignin pathway but also of the tricin pathway, which is embedded in the flavonoid metabolism. It is possible that MYBs from this subgroup have acquired distinct regulatory functions regarding the flavonoid pathway in Arabidopsis and grasses or that tricin biosynthesis is positively controlled by these regulators in grasses because of its involvement in lignification.

Finally, SvMYB74 belongs to subgroup S14, which comprises several regulators of lignification in the context of the Casparian strip formation. In Arabidopsis, AtMYB36 controls the expression of genes encoding the complete machinery required to locally polymerize lignin to form a fine band in the cell wall of endodermal cells, producing the Casparian strip (Kamiya et al. 2015). AtMYB36 is part of a very complex network of 13 MYB transcription factors (from different subgroups) that mediate feedback or feed-forward loops, thus balancing lignification and suberization in Arabidopsis roots (Xu et al. 2022). In rice, three AtMYB36 homologues, OsMYB36a (LOC_Os08g15020), OsMYB36b (LOC_Os02g54520), and OsMYB36c (LOC_Os03g56090), coordinately regulate the temporal and spatial expression of genes essential for Casparian strip formation (Wang et al. 2022). It is noteworthy to observe, however, that subgroup S14 is clearly divided into three clades: (i) a small group of three grass MYBs; (ii) a second clade without any Arabidopsis MYB; and (iii) a third and major clade containing AtMYB36, its rice homologues OsMYB36a/b/c and AtMYB37/38/68/84/87. SvMYB74 clustered within the second clade and was closely related to BdMYB47 (Bradi2g46770) from Brachypodium and to LOC_Os01g49160 and LOC_Os05g48010 from rice. Whereas the former was not functionally characterized, the two rice homologues were linked with different biological processes. LOC_Os01g49160 encodes REGULATOR OF GRAIN NUMBER1 (RGN1), a R2R3-MYB controlling lateral grain formation during panicle development via directly regulation of LONELY GUY (LOG), an enzyme that transforms inactive citokinins into their active forms (Li et al. 2022). LOC_Os05g48010 was mapped as the causative gene underlying the *purple leaf* (*pl*) mutant, possessing colored leaves due to anthocyanin accumulation (Akhter et al. 2019). Loss-of-function of *OsPL* leads to developmental defects, reduced chlorophyl content, and higher levels of anthocyanins, which were accompanied by upregulation of genes from the general phenylpropanoid pathway and from the anthocyanin-specific biosynthetic pathway (Akhter et al. 2019; Xu et al. 2021). Thus, these results demonstrate that at least some of the genes found in this clade within subgroup S14 are involved in the transcriptional regulation of phenylpropanoid metabolism. Our results showed a weaker, although significant, transcriptional activation of all three tested promoters by SvMYB74 when compared to SvMYB18 and SvMYB24. Similar to its closely related homologue in rice OsPL, SvMYB74 might be involved in controlling anthocyanin accumulation in *S. viridis*, maybe playing a minor role in lignification. Nevertheless, the complex regulatory network of SCW deposition involves many players, with distinct functions and involving different transcription factors depending on the organ, cell type and environmental context (Barros et al. 2015).

In conclusion, here we performed a genome-wide characterization of the R2R3-MYB gene family in the C4 grass *S. viridis* and identified four strong candidates to play a role in the regulation of developmental lignification. SvMYB18, SvMYB24 and SvMYB74 were shown to activate the promoters of lignin and tricin biosynthetic genes in transactivation assays using tobacco BY2 protoplasts, which suggests that these transcription factors control grass-specific aspects of lignin deposition. Given that, so far, no transcription factor has been characterized as a specific regulator of tricin biosynthesis, further misregulation of these candidate genes in *S. viridis* might contribute to unravel their function *in planta*.

## Supplementary Information

Below is the link to the electronic supplementary material.

Supplementary file1 Physical distribution of all 132 *SvMYBs* across the 9 chromosomes of *S. viridis* genome and in tandem duplication analysis (PDF 149 KB)

Supplementary file2 Expanded maximum likelihood phylogenetic tree of MYB proteins from different plant species. (PDF 808 KB)

Supplementary file3 RT-qPCR analysis of *SvMYB11* in *S. viridis* tissues/organs contrasting for lignin content (PDF 53 KB)

Supplementary file4 List of primers used in this study. (XLSX 15 KB)

Supplementary file5 RNAseq expression data (FPKM) of SvMYBs in S. viridis retrieved from Martin et al. (2016). (XLSX 20 KB)

Supplementary file6 List of genes co-expressed with selected SvMYBs. Only genes with Pearson Correlation Score above 0.85 are shown. (XLSX 489 KB)
