# Supplementary figures and images for: Genome-wide analysis of the R2R3-MYB family reveals potential regulators of lignin and tricin metabolism in the model grass Setaria viridis
Source: Mol Genet Genomics. 2026 Feb 7;301(1):38. doi: 10.1007/s00438-026-02355-w (PMC12882868; doi:10.1007/s00438-026-02355-w)

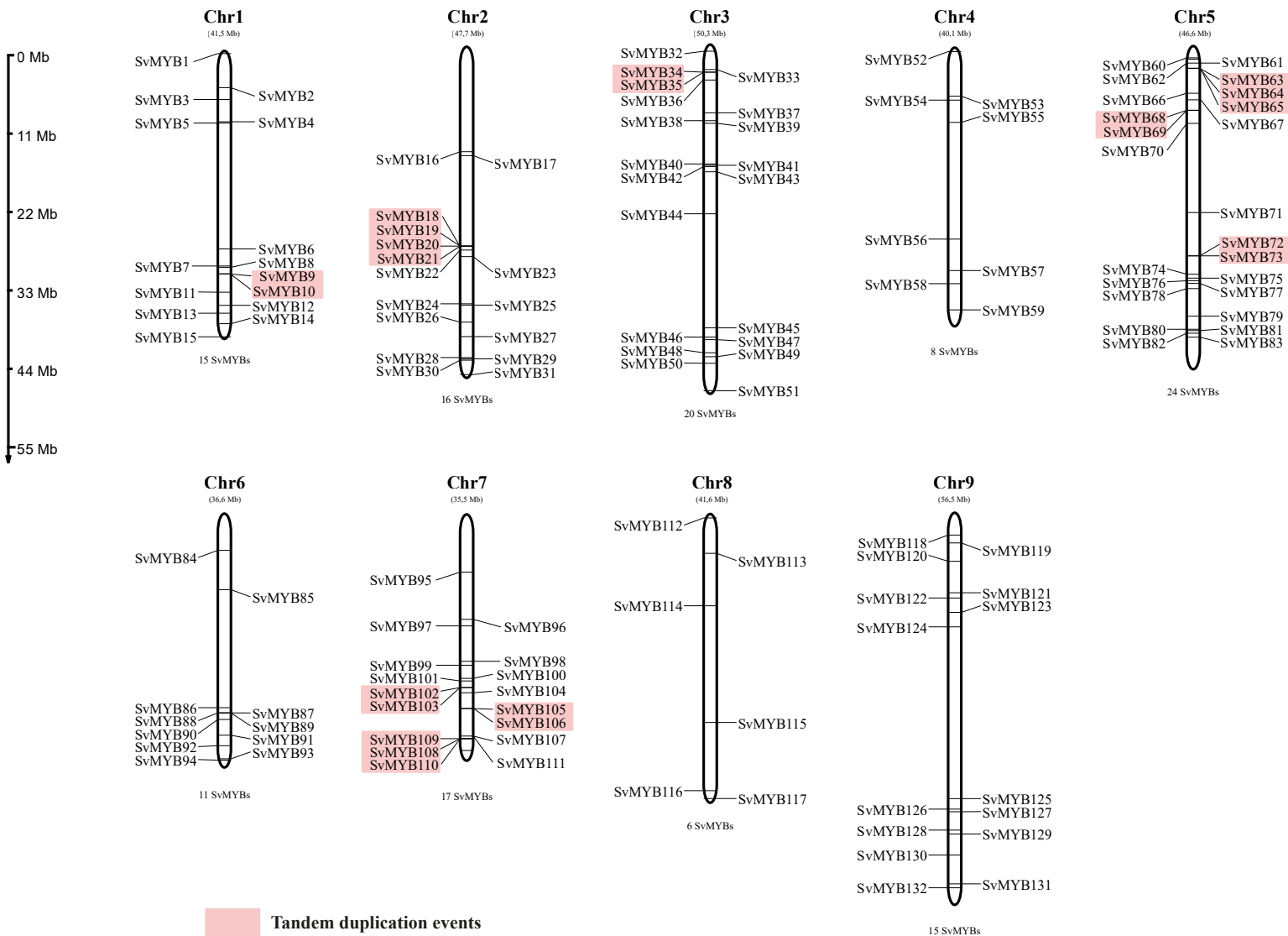

Supplement: Supplementary file 1 — Supplementary file1 Physical distribution of all 132 SvMYBs across the 9 chromosomes of S. viridis genome and in tandem duplication analysis (PDF 149 KB) [file 438_2026_2355_MOESM1_ESM.pdf]

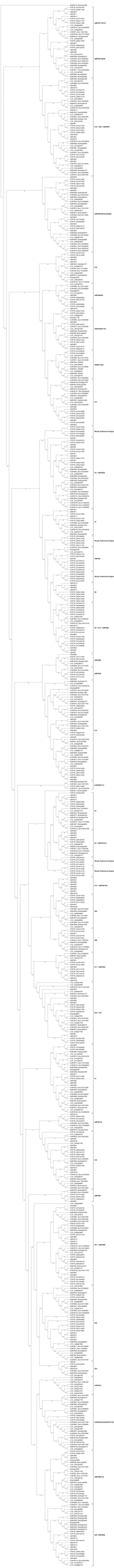

Supplement: Supplementary file 2 — Supplementary file2 Expanded maximum likelihood phylogenetic tree of MYB proteins from different plant species. (PDF 808 KB) [file 438_2026_2355_MOESM2_ESM.pdf]

# SvMYB11

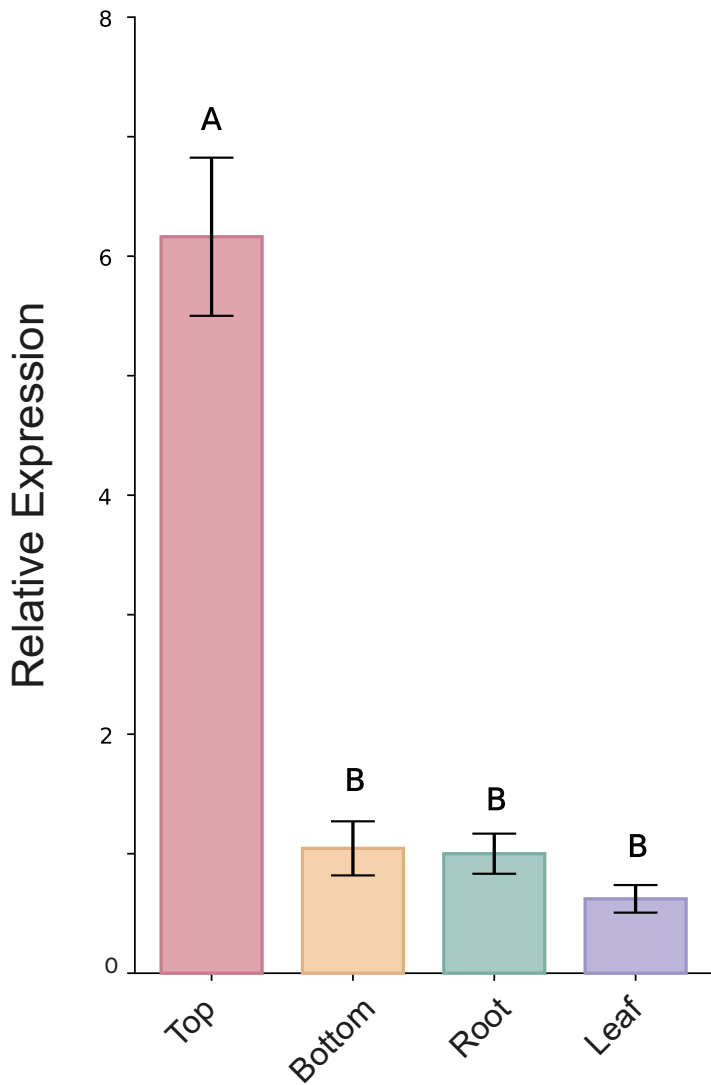

Supplement: Supplementary file 3 — Supplementary file3 RT-qPCR analysis of SvMYB11 in S. viridis tissues/organs contrasting for lignin content (PDF 53 KB) [file 438_2026_2355_MOESM3_ESM.pdf]
